# Ovarian leiomyoma: diagnostic challenges and imaging characteristics in a rare case

**DOI:** 10.1093/bjrcr/uaaf070

**Published:** 2025-12-24

**Authors:** Donna Salam, Maria Hasani, Khalid Ibrahim, Amr Elsisy, Tasnim Keloth, Shareefa Abdulghaffar

**Affiliations:** Radiology Department, Mohammed Bin Rashid University of Medicine and Health Sciences, Dubai, United Arab Emirates; Medical Imaging, Dubai Health, Dubai, United Arab Emirates; College of Medicine, Mohammed Bin Rashid University of Medicine and Health Sciences, Dubai, United Arab Emirates; Medical Imaging, Dubai Health, Dubai, United Arab Emirates; Medical Imaging, Dubai Health, Dubai, United Arab Emirates; Histopathology, Dubai Health, Dubai, United Arab Emirates; Medical Imaging, Dubai Health, Dubai, United Arab Emirates

**Keywords:** ovarian leiomyoma, adnexal mass, pelvic mass, ovarian tumour, spindle cell tumour, computed tomography, magnetic resonance imaging

## Abstract

Ovarian leiomyoma is an extremely rare benign tumour often diagnosed incidentally due to its nonspecific clinical and radiologic features. We present the case of a 48-year-old woman with progressive lower abdominal pain, distention, and genitourinary complaints. Imaging revealed a large complex adnexal mass with both solid and cystic components, seen on ultrasound, CT, and MRI, raising suspicion for a neoplastic process. Despite the use of multiple imaging modalities, definitive diagnosis required histopathological confirmation following surgical resection, which revealed an ovarian leiomyoma with cystic and myxoid degenerative changes. This case underscores the importance of considering ovarian leiomyoma in the differential diagnosis of adnexal masses and highlights the complementary roles of imaging and histopathology in achieving an accurate diagnosis and guiding appropriate management.

## Introduction

Primary ovarian leiomyoma (OL) is an exceptionally uncommon tumour, constituting approximately 0.5%-1% of all ovarian tumours of benign origin. It predominantly affects women aged 20 to 65 years but is most common in the perimenopausal and postmenopausal age groups. Typically, the tumour is small, measuring less than 3 cm in diameter, and is often asymptomatic. However, evidence suggests that OL may exhibit rapid growth during early pregnancy, potentially due to the influence of oestrogen, which may increase the likelihood of encountering larger OLs during this period. In cases where the tumour exceeds 10 cm, some patients may experience abdominal pain and discomfort, and torsion may present as a complication. Ovarian leiomyomas can originate from smooth muscle of hilar blood vessels of the ovary, undifferentiated germ cells, cells within the ovarian ligament, or multipotential cells within the ovarian stroma.^1^^,^^2^ OL is generally unilateral. However, bilateral cases have predominantly been observed in women aged 16 to 25 and have not been documented in individuals over the age of 35.^3^

## Case report

A 48-year-old woman presented to the emergency department with chief complaint of lower abdominal pain and progressive abdominal distention, accompanied by genitourinary symptoms that had developed over the past few months. She had no history of fever, nausea, vomiting, weight loss, or abnormal bowel movements. She had five previous pregnancies culminating in normal vaginal deliveries (NVD). She denies any menstrual complaints, abnormal uterine bleeding (AUB), or discharge. Her menstrual cycles were regular, lasting 6 days with average flow, and her last menstrual period (LMP) was one week before presentation. Her past medical and surgical history was insignificant.

On physical examination, the patient was hemodynamically stable. Her abdomen was distended, roughly the size of an 18-week gravid uterus, with tenderness noted in the lower abdomen. A palpable mass with soft consistency and horizontal mobility was present; the lower limit of the mass could not be felt. Laboratory investigations were deranged, with an elevated red blood cell (RBC) count of 4.95, C-reactive protein (CRP) of 15.1, and LDH of 278. An elevated cancer antigen 125 (CA-125) serum level of 123 U/mL (reference range 0-35 U/mL) was identified, but the levels of carcinoembryonic antigen (CEA), alpha-fetoprotein (AFP), and SCC antigen (SCCA), CA 19-9, CA 15-3, beta HCG were within normal ranges.

**Figure 1.**
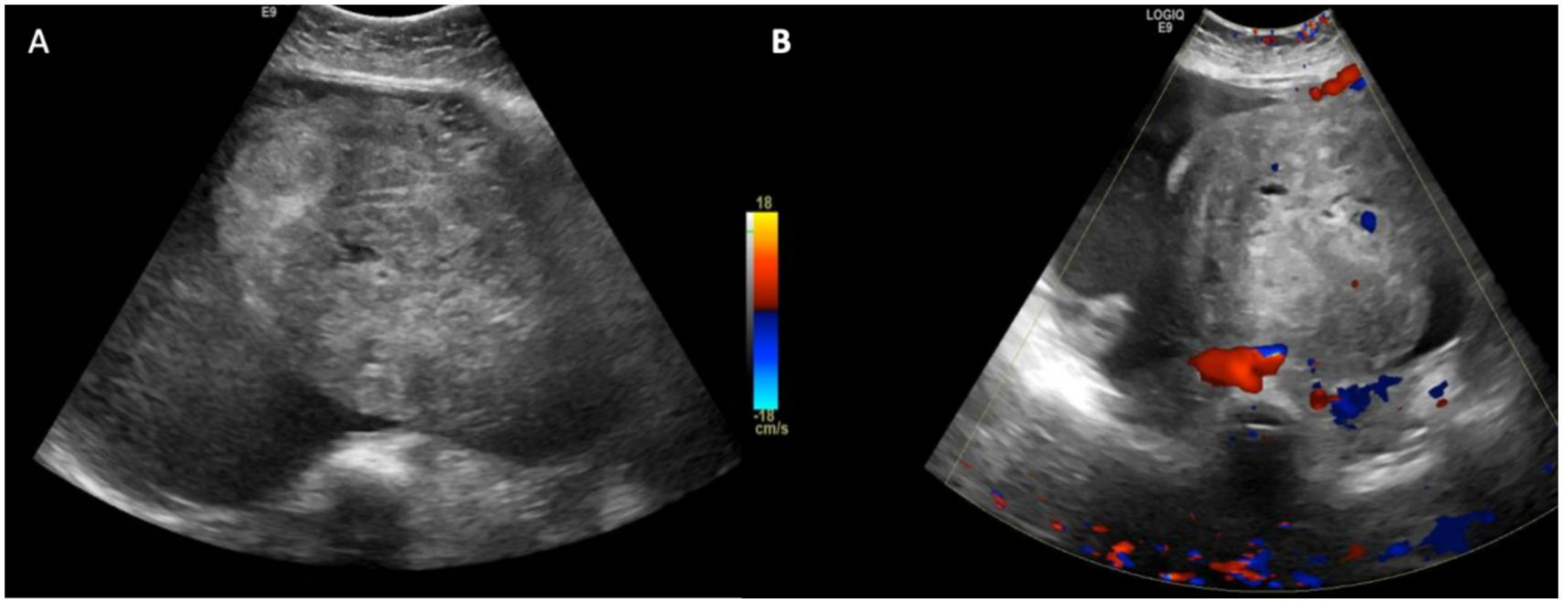
Ultrasound of the abdomen and pelvis showing a very large pelvi-abdominal complex lesion with cystic and vascular solid components, O-RADS US 4 [A, B]. The origin could not be determined from the right adnexa or the uterus.

Radiological imaging was performed, and an abdominal Ultrasound revealed a large complex solid cystic adnexal mass measuring (12.3 × 34 × 24 cm) with a total volume of (3316 ml) filling the abdominopelvic cavity. A large, internal, heterogeneous, predominantly hyper-echoic solid component, showing internal and peripheral vascularity on colour Doppler images, was visualized. No calcification was detected, and there was no ascites. The lesion was classified as O-RADS (Ovarian-Adnexal Reporting and Data System) US 4, indicating an intermediate risk of malignancy (Figure 1).

**Figure 2.**
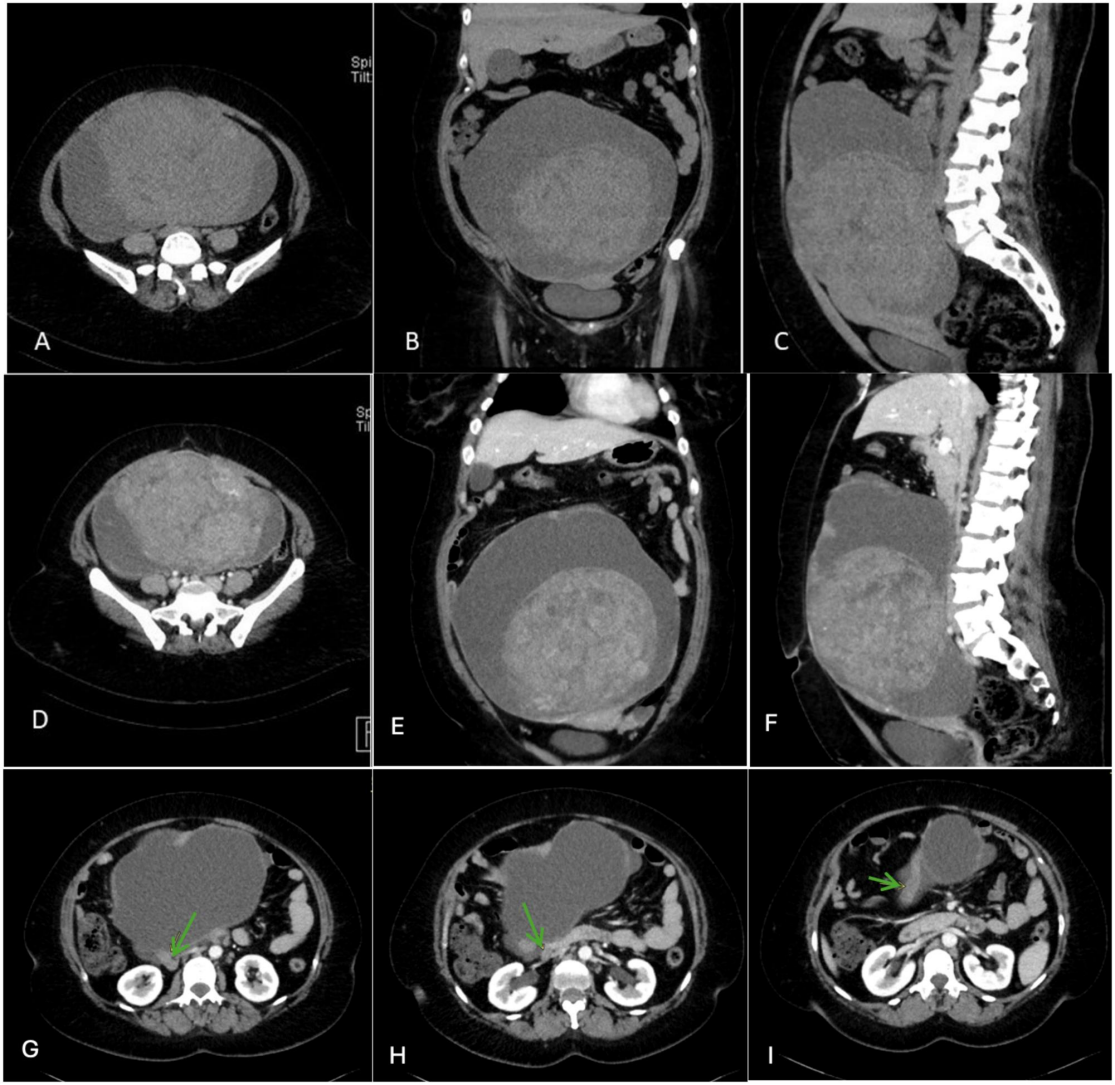
Computed Tomography (CT) of the abdomen and pelvis (non-enhanced axial [A], coronal [B], sagittal [C], and post-contrast axial [D, G, H, I], coronal [E], sagittal [F]) showing a large complex mass with mixed solid and cystic components. The solid component exhibits heterogeneous faint enhancement. Green arrows are pointing towards the tortuous ovarian vein draining the lesion. [G, H, I].

Contrast-enhanced computed tomography (CT) of the abdomen and pelvis was performed, as the mass was suspected to be of either ovarian or uterine origin. A large complex solid and cystic mass extending from the pelvis up to the level of L1 vertebral body, measuring (25 × 14 × 25 cm), was visualized. The solid component of the mass showed heterogeneous attenuation and lobulated margins. There was a subtle enhancement of the feeding vessels passing through the inferior part of the lesion, with no evidence of calcification or internal macroscopic fat densities. Hence, the mass was deemed more likely to originate from the right adnexa rather than the uterus (Figure 2).

**Figure 3.**
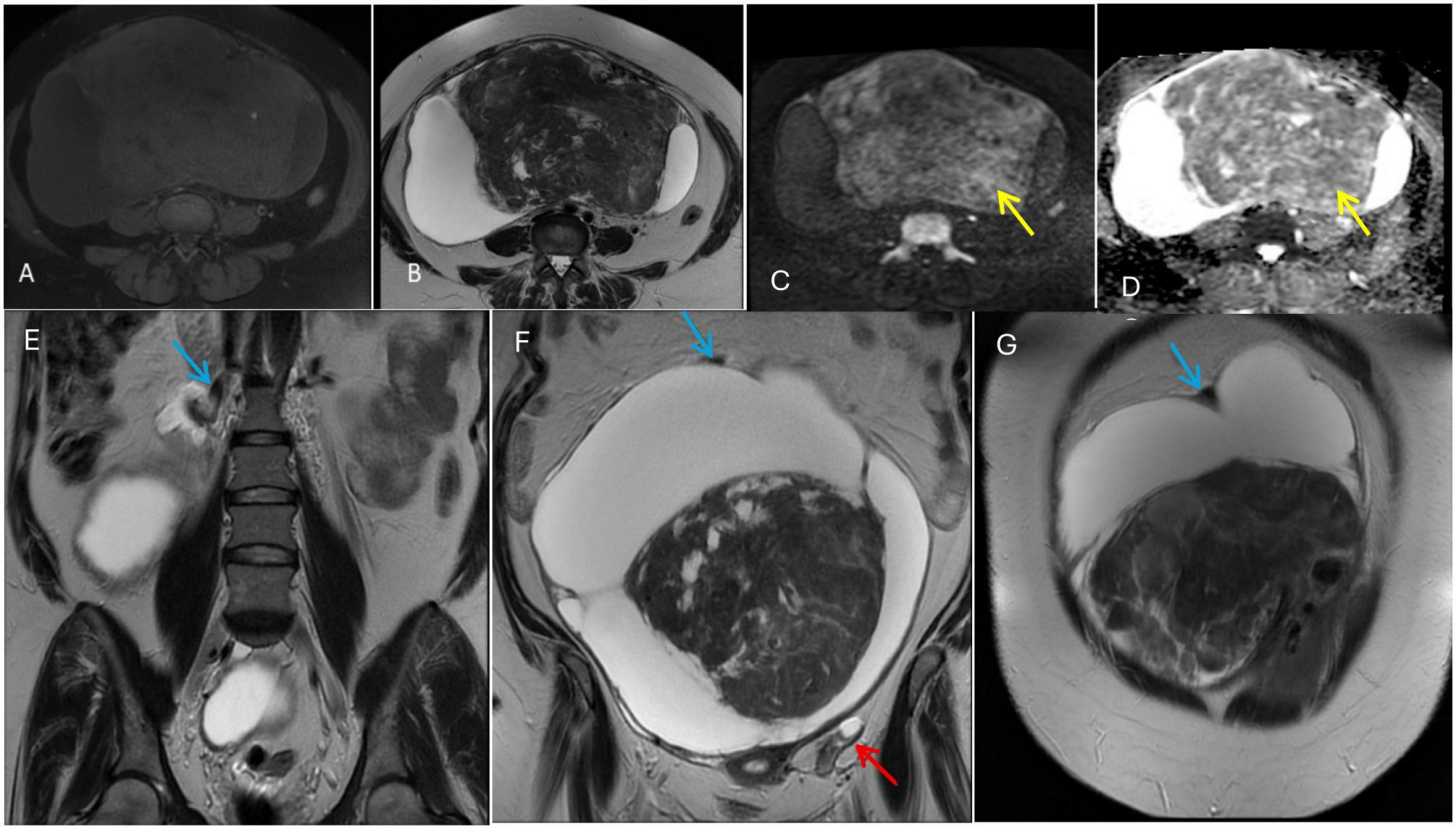
Magnetic Resonance Imaging (MRI) of the pelvis (non-enhanced axial T1 FS [A], axial T2 [B], axial DWI, *b* = 1000 s/mm^2^ [C], axial ADC map [D], coronal T2 [E, F, and G], showing heterogeneous enhancement of the solid component. The solid portion predominantly displays low T2 and intermediate T1 signals, with some areas of bright T2 signal and diffusion restriction (yellow arrows), O-RADS MRI 2. Blue arrows are pointing towards the ovarian vein draining the lesion [E, F, and G], Red arrow indicates the left ovary [F].

**Figure 4.**
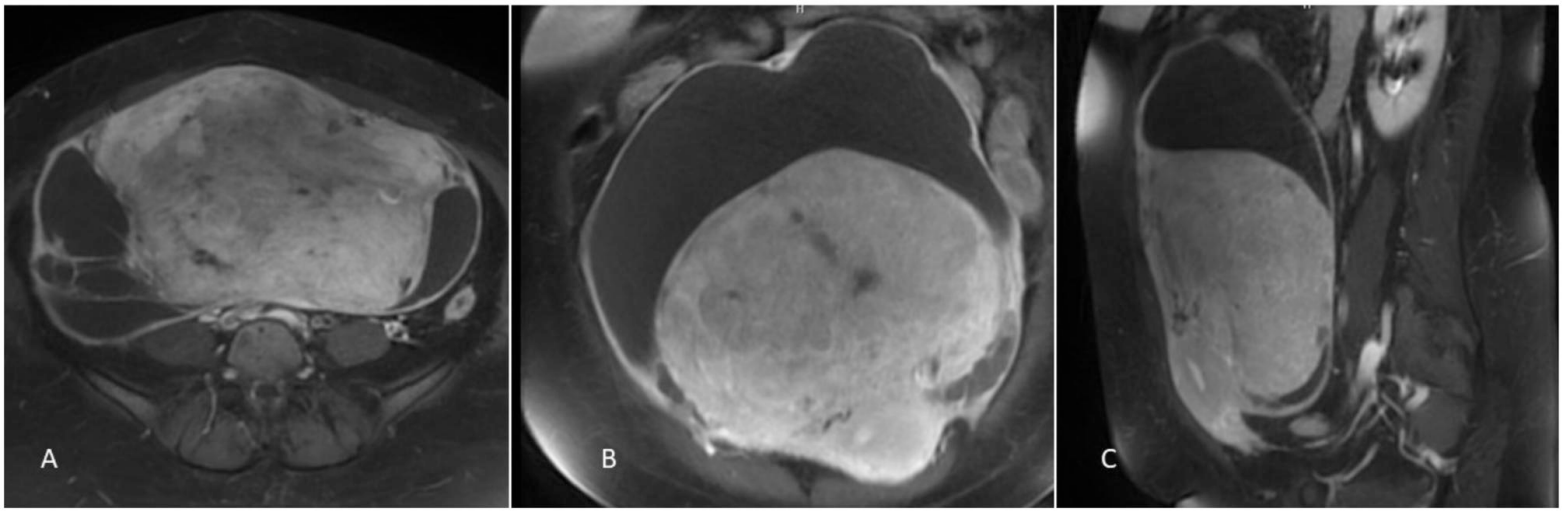
Gadolinium-enhanced Magnetic Resonance Imaging (MRI) of the pelvis (T1 FS axial [A], coronal [B], sagittal [C]) showing a large complex mass with mixed solid and cystic components.

Enhanced Magnetic resonance imaging (MRI) of the pelvis was performed for further characterization and pre-operative planning, and a complex abdominopelvic mass lesion was seen. MRI findings suggested an O-RADS MRI 2 lesion, favouring a benign etiology. It had a thick enhanced wall with multiple septations and contained a large solid component with predominantly low T2 signal and intermediate T1 signal, exhibiting heterogeneous, patchy enhancement. Along with multiple small cystic areas of fluid signal and other cysts of bright T2 signal, diffusion restriction, and post-contrast enhancement. This lesion measured (25 × 14 × 25 cm), causing a mass effect on the uterus anteriorly and the other surrounding structures. It was seen inseparable from the posterofundal uterus with multiple dilated and engorged vessels. The right ovarian vein was identified as draining the lesion (Figures 3 and 4). However, owing to the solid component signal-enhanced pattern as well as possible pedicular attachment to the uterus, a provisional diagnosis of a giant subserous fibroid adherent to the right ovary with larger cystic and fewer myxoid and hyaline degeneration was made.

**Figure 5.**
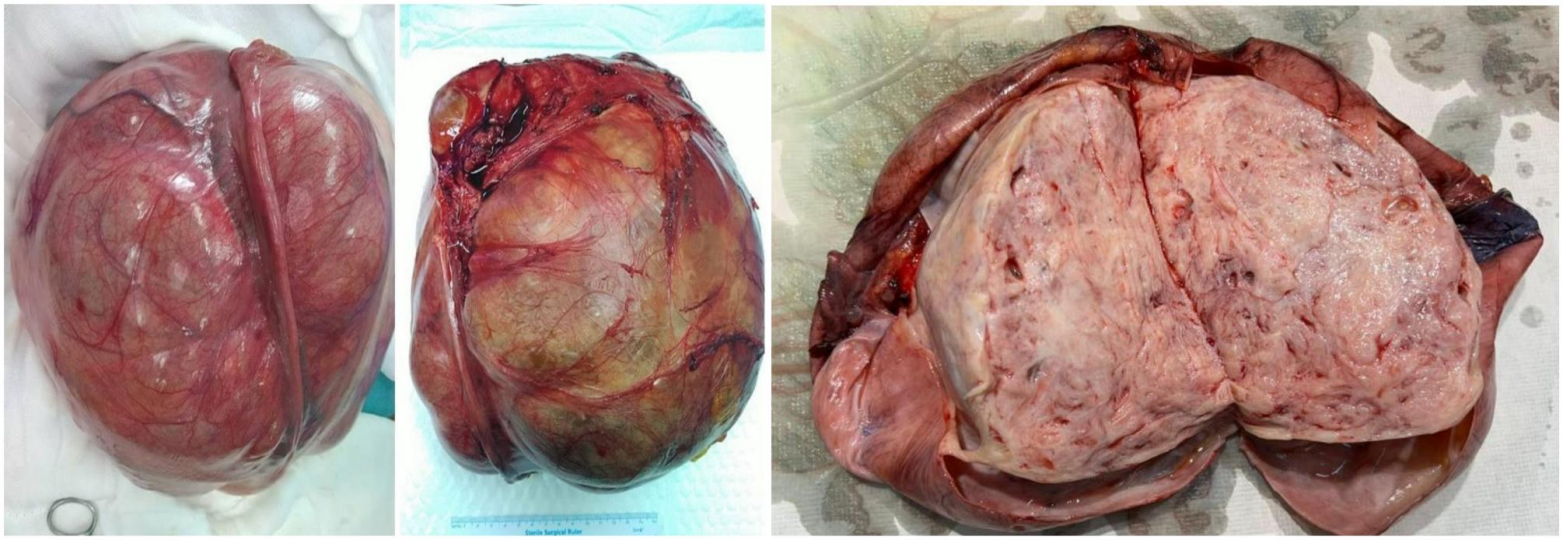
Gross specimen of the excised right ovarian complex cyst, a bosselated pale brown colored mass measuring 28.0 × 27.0 × 17.0 cm, with a glistening serosal surface showing abundant areas of hemorrhage with focal congestion. The surface shows focally attached scanty adipose tissue, showing abundant areas of hemorrhage.

The patient underwent an uncomplicated elective open right salpingo-oophorectomy, left ovarian cystectomy, omentectomy, and right pelvic lymphadenectomy. The intraoperative findings revealed a large right ovarian complex cyst (20 × 20 cm), which was pushing the uterus to the left side and adherent to the right ureter, iliac vessels, appendix, and the posterior wall of the uterus (Figure 5). The right tube was stretched over the large ovarian cyst. It was excised intact following the removal of adhesions. The right ovarian cyst was severely adherent to the posterior uterine wall, while the left ovary had a small cyst (2 cm), which was also excised.

**Figure 6.**
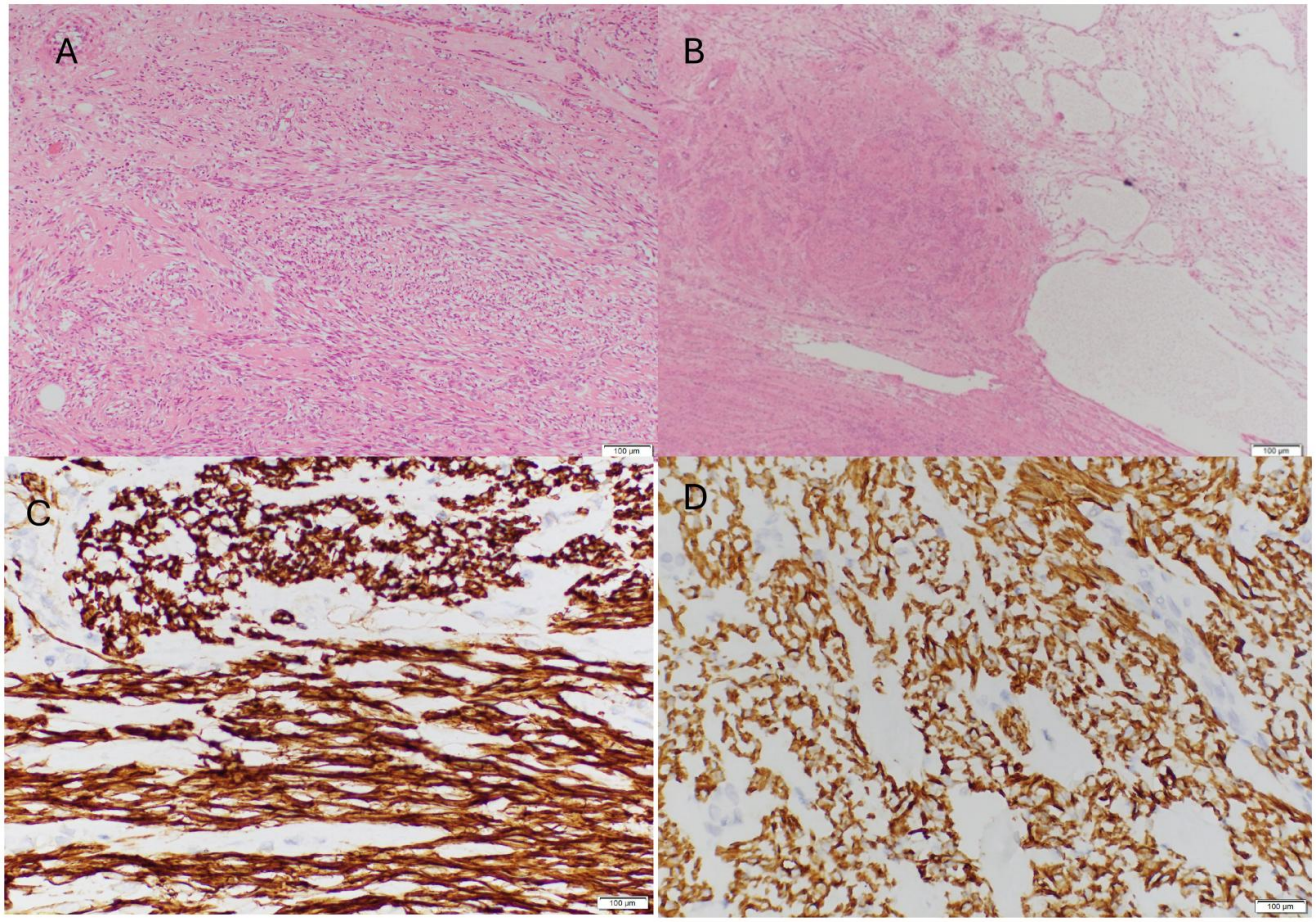
Histopathology: Microscopic findings showing spindle cells arranged in an interlacing and whorled pattern, showing elongated nuclei with blunt ends, focal cystic and myxoid degenerative changes [A and B], and diffuse strong reaction for desmin and caldesmon [C and D].

Histopathology examination of the mass revealed a right ovarian spindle cell tumour with prominent microcystic and macrocystic features along with focal myxoid degenerative changes. The tumour showed no evidence of cellular atypia, mitotic activity, or coagulative necrosis. The histomorphological features and immunoprofile results were consistent with the diagnosis of an Ovarian Leiomyoma (Figure 6).

## Discussion

Primary Ovarian leiomyoma (OL) is an exceptionally uncommon tumour, accounting for only 0.5-1% of all benign ovarian tumours, and is typically solid in composition. Cystic ovarian leiomyoma, a rare subtype of OL, has very few cases documented in the literature, making the presence of extensive cystic degeneration, as in our case, exceedingly rare. OL predominantly affects women aged 20 to 65 years but is most common in the perimenopausal and postmenopausal age groups.^1^^,^^4^

While the majority of OL measure approximately 5.5 cm, the largest recorded case has reached 25 cm. These tumours may exhibit histopathological types, including typical, cellular, and myxoid, and can undergo degenerative changes such as cystic, hyaline, and calcific degeneration.^5^ The leiomyoma in our case had solid and multiple cystic components measuring (25 × 14 × 25 cm). Cystic degeneration is reported in only ∼4% of OLs, making this case notable both for its rarity and size.^6^

Most OLs are predominantly asymptomatic and are typically identified incidentally. In cases where symptoms are present, particularly with a sizable leiomyoma, clinical manifestations may include abdominal pain, a palpable mass, hydronephrosis, elevated CA-125 levels, Meigs syndrome, and even polymyositis. It is important to note that ovarian leiomyomas are frequently misdiagnosed preoperatively as pedunculated uterine myomas, ovarian fibromas, or even ovarian endometriomas.^7^

Ultrasound and MRI are the principal modalities utilized to assess ovarian lesions. On ultrasound, ovarian leiomyomas exhibit an appearance that is quite similar to their uterine counterparts, presenting as heterogeneous, hypoechoic, well-circumscribed masses with minimal or no significant blood flow.^1^^,^^4^

On MRI, these tumours typically present as solid masses, exhibiting either intermediate or low signal intensity on T1-weighted images and low signal intensity on T2-weighted sequences. They demonstrate early enhancement following contrast administration, resembling leiomyomas found in other anatomical locations. Conversely, fibromas or thecomas—benign ovarian tumours with a high prevalence also display low signal intensity on both T1 and T2-weighted MRI and show poor enhancement with contrast.^8^

Specific lesions may reveal areas of high T2 signal, indicative of edematous changes due to ischemia or cystic and myxoid degeneration. Furthermore, encountering an ovarian tumour with a cystic component may prompt consideration of primary ovarian lymphoma in the differential diagnosis.^2^ MRI is valuable in evaluating the ovarian origin of the lesion, and tracing the course of the gonadal vessels can assist in establishing the tumour’s origin. If no distinct ovarian tissue is identified elsewhere, it indicates a primary ovarian origin in most cases.^8^

Preoperative diagnosis is often challenging, especially in identifying the origin of pelvic solid tumours and differentiating them from other similar masses. Cystic degeneration complicates preoperative imaging interpretation and may lead to suspicion of malignancy, particularly when accompanied by elevated CA-125 levels or the presence of ascites, which may necessitate a more invasive surgical approach.^3^ In our case, the cystic architecture, enhancing solid component, and possible pedicular attachment to the uterus made a provisional diagnosis of a giant subserous fibroid with predominant cystic and minor myxoid and hyaline degeneration plausible.

The definitive diagnosis is established through histopathological and immunohistochemical analysis. Both macroscopically and microscopically, ovarian leiomyomas resemble their uterine counterparts. These lesions exhibit positive staining for alpha-smooth muscle actin (α-SMA).^1^ Histopathological examination of OL reveals interlacing bundles of fusiform cells characterized by oval nuclei, exhibiting minimal atypia and mitotic activity akin to uterine leiomyomas. Additionally, hyaline and myxomatous degeneration are frequently observed.^7^^,^^9^ It is essential to differentiate between leiomyoma and leiomyosarcoma, which differ in their degree of mitotic activity. Cases of mitotically active ovarian leiomyoma with a clinically benign course have been documented in the literature.^10^ Prominent microcystic and macrocystic features, along with focal myxoid degenerative change, were seen in our case. This rare secondary change in OL is observed in only a minority of cases.^6^

The synchronous occurrence of ovarian leiomyoma with uterine leiomyoma in 78% of cases indicates similar hormonal stimulation. Primary OL is believed to originate from smooth muscle cells within the ovarian vessels or ligaments. Some researchers propose that these tumours may arise from undifferentiated reproductive cells, cortical smooth muscle cells, or as a result of metaplasia associated with ovarian endometriosis. In contrast, secondary ovarian leiomyomas develop from external tissues in contact with the ovaries. According to Agrawal et al., a primary OL must be entirely contained within the ovary, with no identical lesions in the uterus or other locations.^5^^,^^11^

## Conclusion

This case highlights an exceptionally rare presentation of primary ovarian leiomyoma with extensive cystic degeneration. Although benign with an excellent prognosis, it should be considered in the differential diagnosis of large, complex ovarian masses. Combining imaging modalities and histopathological examination is essential for accurate diagnosis and appropriate surgical management.

## Learning points

- Ovarian leiomyomas are extremely rare tumours, requiring a high index of suspicion for accurate diagnosis.
- Cystic degeneration occurs in less than 4% of ovarian leiomyomas, making this presentation highly uncommon.
- Ovarian leiomyomas are difficult to diagnose radiologically through various imaging modalities, making immunohistopathology necessary for a definitive diagnosis.
- Ovarian leiomyomas carry a very low risk of malignant transformation. Thus, management should prioritize preserving fertility and improving quality of life, avoiding destructive ovarian surgery.

## Data sharing statement

Data described in this study will be publicly available post-publication.
